# Supplementary material for: A key function for microtubule-associated-protein 6 in activity-dependent stabilisation of actin filaments in dendritic spines
Source: Nat Commun. 2018 Sep 17;9:3775. doi: 10.1038/s41467-018-05869-z (PMC6141585; doi:10.1038/s41467-018-05869-z)
Supplement: Supplementary file 1 — Supplementary Information [file 41467_2018_5869_MOESM1_ESM.pdf]

Peris *et al.* 2018
Supplementary Information

Supplementary Figure 1 related to Figure 2

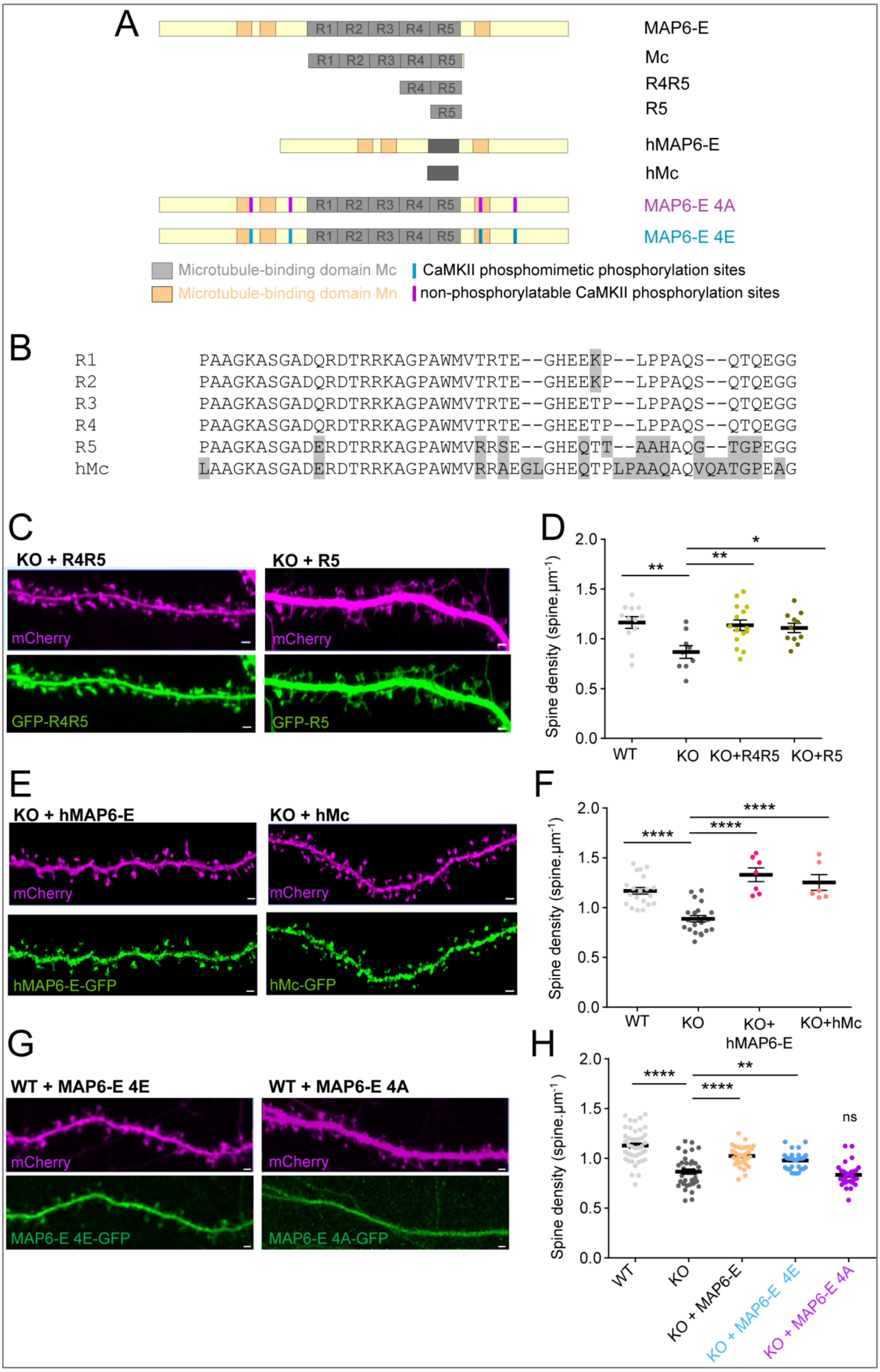

Supplementary Figure 1. Effects of Mc modules, human MAP6 and MAP6 phosphorylation mutants on dendritic spine density

(A) Schematic representation of rodent, human MAP6-E and MAP6-E phosphorylation mutant constructs used in this paper. Rodent MAP6-E, Mc, R4R5 and R5 modules; human hMAP6-E and hMc module; rodent phosphomimic MAP6-E (MAP6-E 4E; blue bars: mutations: S and T to E) or non phosphorylatable MAP6-E (MAP6-E 4A; violet bars: mutations: S and T to A). All cDNAs are in fusion with eGFP cDNA, for a C-terminus fusion except for rodent Mc, R4R5 and R5 with a N-terminal fusion.

(B) Sequence alignment of rat and human Mc modules. The sequences of the five tandemly repeated rat Mc modules (R1 to R5, NP_058900) are aligned with the sequence of the single human Mc module (hMc, NP_149052). Non conserved residues are highlighted in gray. The residues for which the rat R5 sequence differs from R1-R4 are conserved between rat R5 and the single human Mc. Thus the human hMc module is more related to the rat R5 than to the other rat Mc modules.

(C-D) Rescue experiments in MAP6-KO neurons by R4R5 and R5 domain constructs. (C) Confocal images showing representative examples of dendritic segments of MAP6 KO 18 DIV cultured hippocampal neuron transfected with mCherry-expressing vector and eGFP-R4R5 or eGFP-R5 domain constructs. Scale bar: 2 µm. (D) Graphs of dendritic spine density in WT, MAP6 KO neurons and following transfection of MAP6 KO neurons by R4R5 or R5 domain constructs. Data presented as mean ± SEM (*$p<0.05$; **$p<0.01$; one way ANOVA and Tukey's *post hoc* test), n=12, 9, 15 and 11 neurons for WT, KO, KO + R4R5 and KO + R5 respectively.

(E-F) Rescue experiments on MAP6-KO neurons by human hMAP6 and human Mc module. (E) Confocal images showing representative examples of dendritic segments of MAP6 KO 18 DIV cultured hippocampal neuron transfected with mCherry-expressing vector and hMAP6-eGFP or hMc-eGFP domain constructs. Scale bar: 2 µm. (F) Graphs of dendritic spine density in WT neurons, MAP6 KO neurons and after transfection of MAP6 KO neurons with hMAP6 and hMc domain constructs. Data presented as mean ± SEM (**$p<0.01$; ***$p<0.001$ one way ANOVA and Tukey's *post hoc* test), n=20, 21, 7 and 6 neurons for WT, KO, KO + hMAP6-E and KO + hMc respectively.

(G-H) Rescue experiments on MAP6-KO neurons by MAP6-E 4E or MAP6-E 4A constructs. (G) Confocal images showing representative examples of dendritic segments of WT 18 DIV cultured hippocampal neuron transfected with mCherry-expressing vector and MAP6-E 4E or MAP6-E 4A constructs. Scale bar: 2 µm. (H) Graphs of dendritic

spine density in WT and MAP6 KO neurons transfected with mCherry-expressing vector alone or with MAP6-E 4E or MAP6-E 4A constructs. Note that non phosphorylable MAP6-E 4A localizes in the dendritic shaft and is unable to rescue dendritic spine density in MAP6 KO neurons, similar to the MAP6-E ΔMc construct (Fig 2E-F). Data presented as mean ± SEM (* $p<0.05$; ** $p<0.01$; **** $p<0.0001$, one way ANOVA and Sidak's *post hoc test*), n=43, 35, 32, 36 and 28 neurons for WT, KO, KO + MAP6-E, KO + MAP6-E 4E and KO + MAP6-E 4A respectively. For each experiment, neurons were pooled from at least 3 independent cultures.

Human MAP6 only contains one Mc module, whereas Mc modules are present in several copies in rodent MAP6. In this study we used rat MAP6-E, which contains 5 repeats called R1 to R5 (Panel A), R5 being highly similar to the human Mc (Panel B). We investigated the ability of constructs expressing only one or two Mc repeats (R5, R4R5) to rescue the spine density deficit. Both constructs were able, when transfected in MAP6 KO neurons, to significantly increase spine density, from 0.86 ± 0.06 (MAP6 KO) to 1.13 ± 0.05 and 1.03 ± 0.05 spines/µm for R4R5 and R5 respectively (Panel C-D). We next investigated the ability of constructs expressing either the hMAP6-E or the hMc module to correct the spine density deficit (Panel E-F). Both constructs significantly increased spine value density (1.32 ± 0.07 and 1.25 ± 0.08 for hMAP6-E and hMc respectively).
MAP6 contains several CamKII phosphorylation sites known to regulate its affinity for microtubules; moreover, phosphorylation correlates with accumulation of the protein at synapses. We investigated the ability of MAP6-E phosphorylation mutants (Panel A) to correct the spine deficit of MAP6 KO neurons. The mutations converted a set of four serine residues into either glutamic acid (MAP6-E 4E) or alanine (MAP6E-4A), so as to either mimic or prevent phosphorylation by CamKII. As shown in panel G-H, the MAP6-E 4E mutant localized to dendritic spines and was able to increase the abnormally low spine density of MAP6 KO neurons (0.865 ± 0.025 spines/µm) to a value similar to that of MAP6 KO neurons rescued by MAP6-E, or of WT neurons (0.978 ± 0.013; 1.025 ± 0.017 and 1.12 ± 0.025 spines/µm, for MAP6 KO neurons rescued by MAP6E-4E or by MAP6-E and for WT neurons, respectively). In contrast, the MAP6-E 4A mutant remained restricted to the dendritic shaft and was unable to restore the spines of MAP6 KO neurons (Panel G-H, 0.833 ± 0.022 and 0.865 ± 0.026 spines/µm for MAP6 KO neurons expressing MAP6-E-4A and MAP6 KO neurons, respectively).

Supplementary Figure 2 related to Figure 3

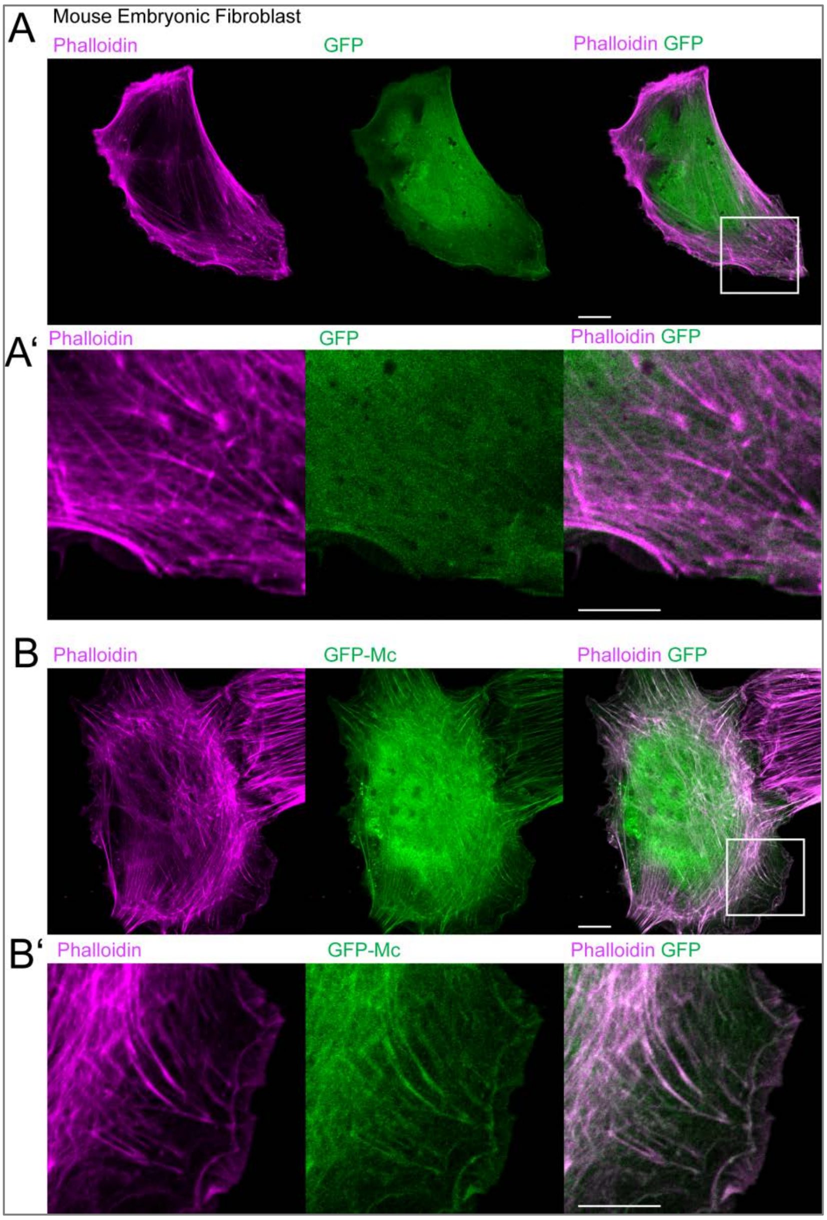

Supplementary Figure 2. Sub-cellular localisation of GFP or GFP-Mc in fibroblasts.

Confocal images showing Mouse Embryonic Fibroblasts expressing either GFP or GFP-Mc and co-stained with phalloidin (red) to label actin filaments.

(A) GFP labelling is diffuse throughout the cell, even at the cell edges where actin filaments form stress fibres and lamellipodia. (A') Higher magnification view of the insert box shown in A.

(B) GFP-Mc showing a diffuse pattern in the center of the cell and a colocalization with actin stress fibres and lamellipodia at the edges of the cell. (B') Higher magnification view of the insert box shown in B.

Scale bars: 10 µm.

Supplementary Figure 3 related to Figure 5 and 6

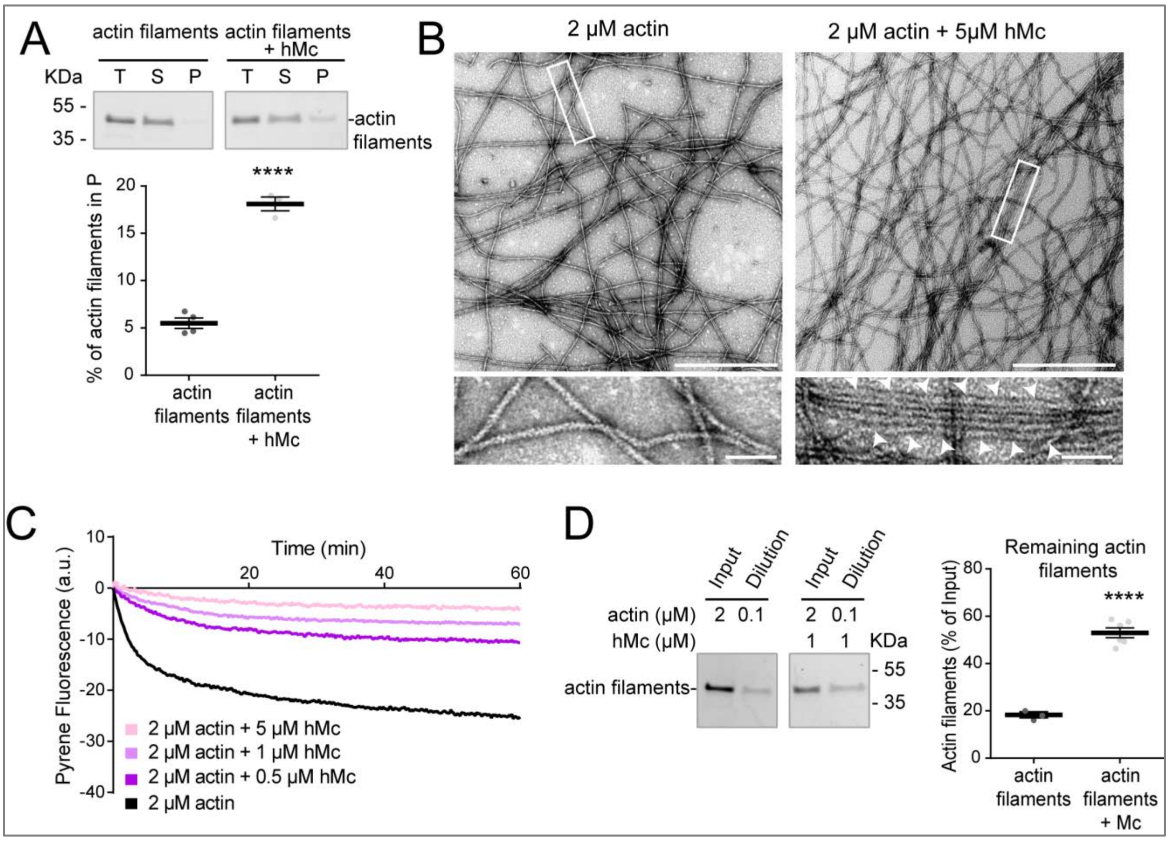

Supplementary Figure 3. Human Mc module effects on actin dynamics and organisation

(A) Actin filaments low speed co-sedimentation assay. Actin filaments (2 µM) were incubated alone or with hMc module (1 µM), centrifuged at 15,000 *g* and total (T), supernatant (S) and pellet (P) fractions were analyzed on SDS/PAGE and actin was detected by Stain-Free technology in the gel. Graph shows the quantification of actin bundles (in pellet). Note that almost 20% of actin filaments sedimented in the presence of hMc, against 5% without hMc module. Data presented as mean ± SEM (**p<0.01, ****p<0.0001; Student's t-test). n=4 and 3 gels for actin alone and actin + hMc respectively, of 2 independent experiments.

(B) Actin filaments (2 µM) formed alone or in the presence of hMc module (5 µM) were imaged by negative staining and electron microscopy. Scale bar: 500 nm. Lower panels: higher magnifications of the respective boxed regions. Note that the hMc module also induces a straightening of actin filaments which are organized in tight arrays with periodic striations (white arrowhead) similar to those observed with Mc modules. Scale bar: 50 nm.

(C) Actin depolymerisation induced by buffer dilution. Mean time course of actin filaments and hMc decorated actin filaments depolymerisation. After 1 hour incubation of 40% pyrene labelled actin filaments (2 µM) alone or in the presence of hMc module (0.5 µM, 1µM and 5

µM), all samples were diluted to a final concentration of 0.1 µM of actin (alone or with 0.5 µM, 1 µM or 5 µM of hMc) and pyrene fluorescence was measured over time.

(D) High speed sedimentation assay of remaining actin filaments resistant to buffer dilution. Actin filaments (2 µM) alone or in the presence of hMc module (1 µM) were incubated for 20 minutes in a buffer containing 0.1 µM actin (alone or with 1 µM of hMc). Samples were centrifuged at 100,000 *g* and pellets (before dilution: input, or after dilution) were analyzed by SDS/PAGE and actin was revealed by Stain-Free system. Graph shows the percentage of remaining actin filaments after buffer dilution. Data presented as mean ± SEM (****p<0.0001, Student's t-test). n=3 and 6 gels for actin alone and actin + hMc of 2 independent experiments.

Supplementary Figure 4

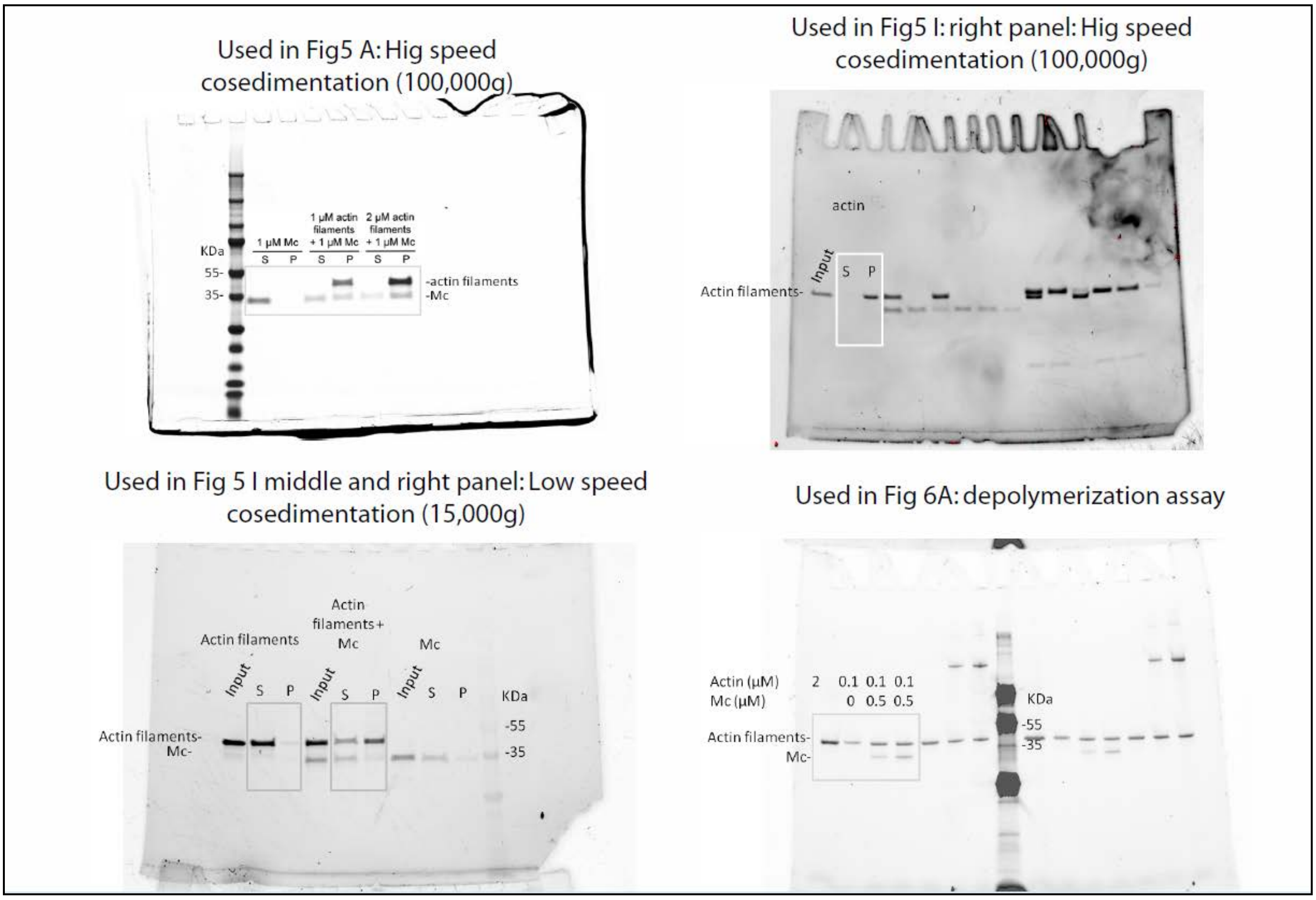

Pictures of SDS PAGE gels revealed by Stain-Free system and used to built the figures in main manuscript.
