# Supplementary material for: A key function for microtubule-associated-protein 6 in activity-dependent stabilisation of actin filaments in dendritic spines
Source: Nat Commun. 2018 Sep 17;9:3775. doi: 10.1038/s41467-018-05869-z (PMC6141585; doi:10.1038/s41467-018-05869-z)
Supplement: Supplementary file 3 — Description of Additional Supplementary Files [file 41467_2018_5869_MOESM3_ESM.pdf]

Description of Additional Supplementary Files

File Name: Supplementary Movie 1
Description: Acceptor Photobleaching FRET (ap FRET) analysis of 2 DIV cultured hippocampal neuron transfected with either GFP (left) or MAP6-E GFP (right) constructs and stained for actin filaments (TRITC-phalloidin). Video represent Donor images (GFP tagged) taken before (pre-) and after (post-) acceptor photobleaching. For better visualization of the donor fluorescence intensity, a pseudo-colour lookup table (LUT) was applied to pre- and post-bleach images. Arrowheads show acceptor bleached growth cones. Scale bar: 20 μm.
